# Retrospective Cohort Analysis of Chest Injury Characteristics and Concurrent Injuries in Patients Admitted to Hospital in the Wenchuan and Lushan Earthquakes in Sichuan, China

**DOI:** 10.1371/journal.pone.0097354

**Published:** 2014-05-09

**Authors:** Xi Zheng, Yang Hu, Yong Yuan, Yong-Fan Zhao

**Affiliations:** Department of Thoracic Surgery, West China Hospital, Sichuan University, Chengdu, Sichuan Province, China; The Ohio State University, United States of America

## Abstract

**Background:**

The aim of this study was to compare retrospectively the characteristics of chest injuries and frequencies of other, concurrent injuries in patients after earthquakes of different seismic intensity.

**Methods:**

We compared the cause, type, and body location of chest injuries as well as the frequencies of other, concurrent injuries in patients admitted to our hospital after the Wenchuan and Lushan earthquakes in Sichuan, China. We explored possible relationships between seismic intensity and the causes and types of injuries, and we assessed the ability of the Injury Severity Score, New Injury Severity Score, and Chest Injury Index to predict respiratory failure in chest injury patients.

**Results:**

The incidence of chest injuries was 9.9% in the stronger Wenchuan earthquake and 22.2% in the less intensive Lushan earthquake. The most frequent cause of chest injuries in both earthquakes was being accidentally struck. Injuries due to falls were less prevalent in the stronger Wenchuan earthquake, while injuries due to burial were more prevalent. The distribution of types of chest injury did not vary significantly between the two earthquakes, with rib fractures and pulmonary contusions the most frequent types. Spinal and head injuries concurrent with chest injuries were more prevalent in the less violent Lushan earthquake. All three trauma scoring systems showed poor ability to predict respiratory failure in patients with earthquake-related chest injuries.

**Conclusions:**

Previous studies may have underestimated the incidence of chest injury in violent earthquakes. The distributions of types of chest injury did not differ between these two earthquakes of different seismic intensity. Earthquake severity and interval between rescue and treatment may influence the prevalence and types of injuries that co-occur with the chest injury. Trauma evaluation scores on their own are inadequate predictors of respiratory failure in patients with earthquake-related chest injuries.

## Introduction

In 2008, the devastating Wenchuan earthquake hit China’s Sichuan province and led to 69,181 deaths, 18,522 missing and 374,171 injured. Five years later, another violent earthquake hit Sichuan, this time in the Lushan area, leading to at least 196 deaths, 21 missing, and 11,470 injured. The Wenchuan earthquake was rated as 8.0 on the Richter scale, with a maximum intensity of XI on a seismic intensity scale of I–XII defined by the Chinese State Bureau of Quality and Technical Supervision. The Lushan earthquake was rated as 7.0 on the Richter scale with a maximum intensity of IX. Both earthquakes occurred in geologically and demographically similar areas, providing an unusual opportunity to compare the prevalence and types of casualties in two earthquakes differing primarily in seismic intensity.

Previous studies have reported that chest injuries account for only a small proportion of all earthquake-related injuries, and that most patients with chest injuries present with other, concurrent types of injuries that can significantly affect clinical outcomes [1]–[3]. These studies have not, however, addressed the question of whether the characteristics of chest injuries or co-occurring injuries vary with earthquake intensity. Addressing this question ideally requires examining two earthquakes that differ in intensity but that affect similar geologic areas with similar types of populations and distributions, and in which the casualties were treated at similar medical facilities. West China Hospital of Sichuan University is roughly equidistant from the epicenters of both earthquakes and was unaffected; it provided medical treatment to more trauma patients in both disasters than other hospitals, altogether 4,092 patients in the Wenchuan earthquake and 400 in the Lushan earthquake. Thus the data collected at West China Hospital on patients treated in both earthquakes provide a unique opportunity to examine the effects of seismic intensity on injury characteristics. Such comparisons may help predict the types of injuries likely to occur in earthquakes of given severity and help administrators allocate limited medical resources appropriately.

We also hoped to take advantage of our data set from two earthquakes in order to verify the efficacy of three types of trauma scoring systems for predicting respiratory failure in patients with chest injuries, all based on the the Abbreviated Injury Scale 2005 (AIS2005): the Injury Severity Score (ISS), the New Injury Severity Score (NISS) and the Chest Injury Index (CII). Our analysis of patients in the Wenchuan earthquake suggested that NISS was superior to the other indices [1], [4], so we wished to verify that finding using data from the Lushan earthquake.

## Materials and Methods

The Institutional Review Board of West China Hospital approved the protocol for this retrospective study before it was undertaken; informed consent from participants was ruled unnecessary because we retrospectively analyzed data routinely collected from all inpatients and we analyzed the data anonymously.

Patients from earthquake-affected areas were transported either directly by helicopter from the scene to West China Hospital, or by car or helicopter from field hospitals. All patients admitted to our hospital with possible chest disorders were examined by two thoracic surgeons. Diagnosis of chest injury was made based on physical examination, chest X-ray, computed tomography (CT), fibrobronchoscopy or diagnostic pleuracentesis. All patients with chest injuries were included in our study. Since injuries of the thoracic vertebrae, scapula, and clavicle were treated by orthopedic surgeons, patients with only these injuries but no concurrent chest injury were excluded from our study. Simple contusion of the chest wall and injuries unrelated to the earthquakes, such as spontaneous pneumothorax, were also excluded.

Several items of information were collected from all patients after admission, including age, sex, location where the injury occurred, whether the injury occurred during the primary shock or during aftershocks, the cause and type of injury, diagnosis, duration of mechanical ventilation, and treatment outcome (recovery, deterioration or death). The procedures used to collect these data and update them after the Wenchuan earthquake have been described [4]; the data from patients in the Lushan earthquake were up-dated through 20 July 2013.

Causes of injuries were categorized based on previous studies of earthquakes and reports from the U.S. Centers for Disease Control and Prevention [5]: injuries due to falls, being buried, or being accidentally struck, or other injuries, such as burns, cuts, motor vehicle crashes, and delayed effects of injuries. The cause of injury of patients with multiple injuries were classified based on the cause of the clinically most important injury; for example, patients with multiple injuries due primarily to prolonged burial were classified as having injury due to burial.

In order to estimate the seismic intensity experienced by patients at the time of injury, we used Richter scale data and intensity distribution maps of both earthquakes generated based on Xu et al. [6] and data from the China Earthquake Networks Center. Seismic intensity assesses the intensity of ground shaking and consequences of an earthquake. Seismic intensity usually weakens with increasing distance from the epicenter, except for certain locations where this relationship does not hold. The locations where patients sustained injuries were located on this map and used to explore possible relationships between seismic intensity and the causes and types of injuries. We included in this analysis only patients for whom we obtained reliable information about where they had sustained their injuries.

Chest injuries were assessed using the ISS, NISS, and CII scales as described [4]. Patients who were mechanically ventilated for more than 24 h, either invasively or noninvasively, were diagnosed with respiratory failure.

Data on causes and types of injury in patients from both earthquakes were analyzed using SPSS 17.0 (IBM, Chicago, IL). Differences in continuous variables between patients from the two earthquakes were assessed for statistical significance using Student’s t test or the Mann-Whitney U test. Differences in dichotomous variables were analyzed using the chi-squared test or Fisher’s exact test. In order to compare the accuracy with which ISS, NISS and CII predict respiratory failure, we calculated receiver operating characteristics (ROC) curves and cut-off values using Medcalc 11.4.2.0 software. A two-sided p<0.05 was considered significant.

## Results

In the Wenchuan earthquake, 1,856 patients were admitted to West China Hospital, and 184 (9.9%) were diagnosed with chest injuries [4], [7]. All these patients were admitted after the primary shock; unfortunately patient data were not collected in the wake of the aftershocks. After excluding one woman diagnosed with spontaneous diaphragmatic hernia, we retained 183 in the final analysis. Of these, 165 (90.16%) showed improved and were discharged from our hospital within two months after the quake. A total of 28 patients (15.3%) were diagnosed with chest injuries without concurrent injuries. Thoracic surgical procedures, including closed thoracic drainage, were performed in 69 patients (37.7%). Six of the 183 (3.28%) died, accounting for 18.18% of all the 33 deaths in our hospital in the Wenchuan earthquake [7]. In the Lushan earthquake, 81 of 325 inpatients (24.9%) were diagnosed with chest injuries. Of these, we excluded 7 patients whose injuries were not earthquake-related and 2 patients with simple contusion of the chest wall, leaving 72 patients (22.2%) in the final analysis. None of the patients in our cohort presented with chest injuries exclusively; all had at least one additional injury on admission. Of the total 72 chest injury patients, 67 were injured during the primary shock, and 5 during aftershocks. By 20 July 2013, 60 (83.3%) had shown improvement and had been discharged from our hospital. By this date, no deaths among the patients admitted to our hospital were declared after the earthquake. A total of 15 patients (20.83%) received thoracic surgical procedures.

The trauma patients from both earthquakes had a similar age (p = 0.316) and gender composition (p = 0.397). We categorized each patient by his or her principal type of injury, and found that the frequencies of most types of injury differed significantly between the two earthquakes (Table 1). The frequency of “other” injuries could not be compared because of inadequate sample size. Injuries due to being accidentally struck were among the most frequent injuries in both earthquakes; other frequent causes of injury were being buried (Wenchuan) or falling (Lushan).

**Table 1:** Comparison of the causes of chest injury in patients admitted to our hospital in the Wenchuan and Lushan earthquakes.

Cause of injury^a^	Wenchuan, n (%)	Lushan, n (%)	p*
Being buried	21 (11.5)	1 (1.4)	0.010
Being accidentally struck	143 (78.1)	39 (54.2)	<0.001
Falling	18 (9.8)	30 (41.7)	<0.001
Other	1 (0.5)	2 (2.8)	0.193

a Patients with injuries reflecting multiple causes were classified according to the cause of their principal injury.

*Based on the Pearson chi-squared test or Fisher’s exact test.

Among patients with chest injuries in both earthquakes, the most frequent diagnoses were rib fractures and pulmonary contusions (Table 2). Rib fracture was more frequent in the Wenchuan earthquake, while pulmonary contusion predominated in the Lushan disaster. The frequencies of hemothorax, pneumothorax and sternum fracture differed significantly between the two earthquakes.

**Table 2:** Comparison of the prevalence of types of chest injury among patients admitted to our hospital in the Wenchuan and Lushan earthquakes.

Chest injury type	Wenchuan, n (%)	Lushan, n (%)	p*
Rib fracture	161 (88.0)	39 (54.2)	<0.001
Pulmonary contusion	117 (63.9)	57 (79.2)	0.019
Hemopneumothorax	39 (21.3)	9 (12.5)	0.105
Hemothorax	33 (18.0)	1 (1.4)	<0.001
Pneumothorax	19 (10.4)	2 (2.8)	0.047
Fracture of sternum	4 (2.2)	7 (9.7)	0.020
Diaphragmatic hernia	2 (1.1)	0 (0.0)	1.000
Thoracic duct injury	1 (0.5)	0 (0.0)	1.000
Traumatic asphyxia	1 (0.5)	0 (0.0)	1.000
Mediastinal emphysema	0 (0.0)	2 (2.8)	0.079
Mediastinal effusion	0 (0.0)	1 (1.4)	0.282

*Based on the Pearson chi-squared test, continuity-corrected chi-squared test or Fisher’s exact test.

Injuries co-occurring with chest injuries were classified into 1 of 6 regions and compared between the two earthquakes (Table 3). Patients with chest injuries presented with concurrent head and spine injuries more often in the Lushan earthquake than in the Wenchuan earthquake.

**Table 3:** Comparison of the prevalence and types of injuries co-occurring with chest injury in patients admitted to our hospital after the Wenchuan and Lushan earthquakes.

Concurrent injury type^a^	Wenchuan, n (%)	Lushan, n (%)	p*
Brain and skull	33 (18.0)	23 (31.9)	0.016
Face and neck	8 (4.4)	5 (6.9)	0.600
Abdomen	19 (10.4)	13 (18.1)	0.096
Spine and spinal cord	51 (27.9)	39 (54.2)	<0.001
Extremities^b^	113 (61.7)	35 (48.6)	0.056
External injuries	64 (35.0)	34 (47.2)	0.070

a Some patients contained concurrent injuries of multiple types; those patients therefore appear multiple times in the table.

b Extremities include the upper and lower limbs, clavicle, scapula, bony pelvis and hip.

*Based on the Pearson chi-squared test or continuity-corrected chi-squared test.

Next we explored whether chest injury frequency and characteristics were associated with earthquake severity based on data from both earthquakes. We could perform this analysis on only 177 patients because we did not have reliable information about where other patients sustained their injuries. Given this limited sample size, and given the fact that structures in the areas affected by both earthquakes were built to withstand earthquakes up to an intensity of VII (Ministry of Housing and Urban-Rural Development of the People’s Republic of China, code of aseismic design of buildings), we divided seismic intensities into two categories, IV–VII and VIII–XI. Types of injury varied with seismic intensity (Table 4): the frequency of being unintentionally struck increased with greater intensity, whereas the frequency of falling decreased. In contrast, the frequencies of different types of chest injury were similar for the two intensity categories (Table 5).

**Table 4:** Relationships between seismic intensity and cause of injury in patients admitted to our hospital after the Wenchuan and Lushan earthquakes.

Cause of injury, n (%)	Seismic intensity		p*
	IV–VII	VIII–XI	
Being buried	3 (4.1)	6 (5.8)	<0.001
Being accidentally struck	38 (51.4)	86 (83.5)	
Falling	31 (41.9)	10 (9.7)	
Other	2 (2.7)	1 (1.0)	

*Based on the likelihood ratio of the chi-squared test.

**Table 5:** Relationships between seismic intensity and the prevalence of types of chest injury in patients admitted to our hospital after the Wenchuan and Lushan earthquakes.

Chest injury type, n (%)^a^	Seismic intensity		p*
	IV–VII	VIII–XI	
Rib fracture	52 (39.1)	86 (46.2)	0.267
Pulmonary contusion	55 (41.4)	61 (32.8)	
Hemopneumothorax	11 (8.3)	21 (11.3)	
Hemothorax	4 (3.0)	4 (2.2)	
Pneumothorax	5 (3.8)	11 (5.9)	
Fracture of sternum	6 (4.5)	3 (1.6)	

a Some patients suffered from more than one type of chest injury and therefore appear multiple times in this table. One patient with diaphragmatic hernia, one with thoracic duct injury, two with mediastinal emphysema and one with mediatinal effusion were not included in the analysis.

*Based on the likelihood ratio chi-squared test.

**Figure 1:**
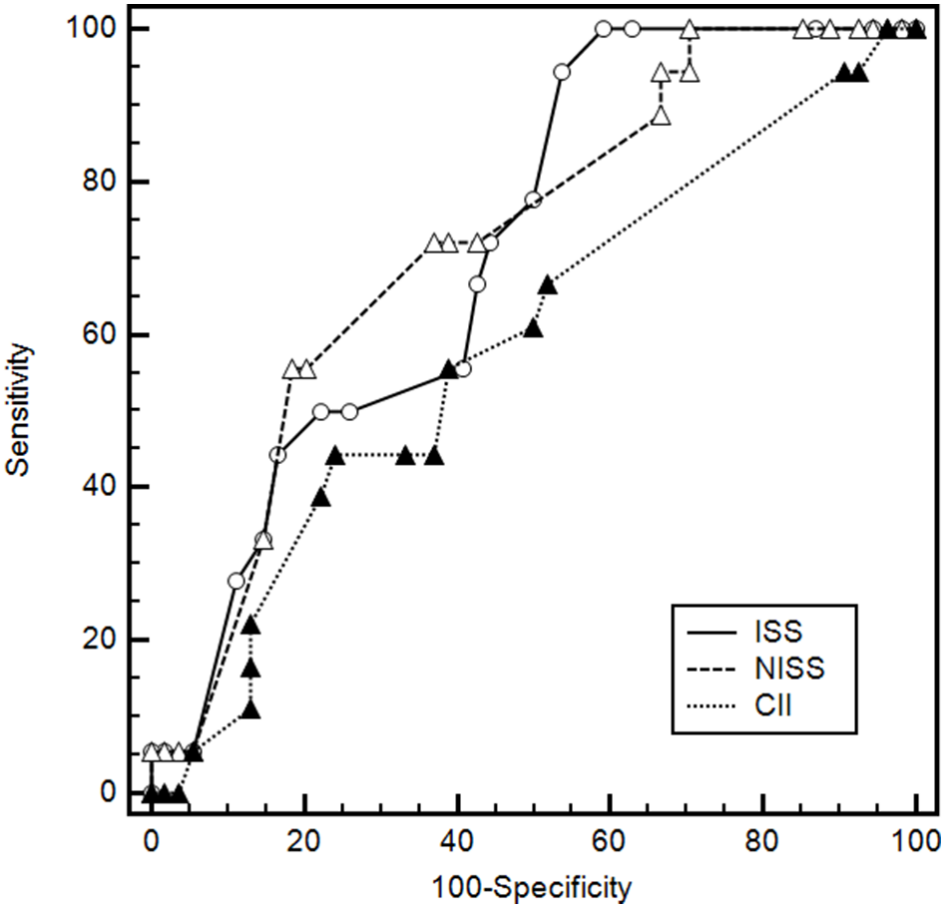
Receiver operating characteristic (ROC) curves of different trauma scoring systems for predicting respiratory failure in patients admitted to our hospital in the Lushan earthquake. The solid line indicates the ROC curve for the Injury Severity Score (ISS); the dashed line, the curve for the New Injury Severity Score (NISS); and the dotted line, the curve for the Chest Injury Index (CII) [4].

In the Wenchuan earthquake, 38 inpatients with chest injuries (20.7%) suffered from respiratory failure [4]. In the Lushan disaster, 18 of the 72 inpatients with chest injuries (25.0%) developed respiratory failure. Building on our previous work assessing the ability of the ISS, NISS and CII scales to predict respiratory failure in chest injury patients in the Wenchuan earthquake, we performed the same analysis on Lushan patients. We calculated the area under ROC curves and estimated 95% confidence intervals (95%CI) for all scales (Figure 1 and Table 6). Although NISS was significantly more accurate than the two other scales in our analysis of Wenchuan chest injury patients [4], all three scales performed similarly poorly for Lushan chest injury patients.

**Table 6:** Comparison of the ability of conventional injury severity scores to predict respiratory failure in patients admitted to our hospital from the Lushan earthquake.

Scoring system	Cut-off value	Sensitivity (%)	Specificity (%)	AUC	95%CI
ISS	14	100.0	40.7	0.713^a^	0.594–0.813
NISS	28	55.6	81.5	0.720	0.601–0.819
CII	18	44.4	75.9	0.590^b^	0.468–0.705

Abbreviations: AUC, area under the ROC curve; CII, chest injury index; ISS, injury severity score; NISS, new injury severity score; 95%CI, 95% confidence interval.

a NISS vs. ISS, p = 0.880.

b NISS vs. CII, p = 0.136.

## Discussion

Earthquakes are major geological disasters that cause tremendous human and economic losses around the world [8], [9]. However, relatively little is known about how earthquake factors such as seismic intensity affect the prevalence and types of casualties. Here we examine the prevalence and causes of different types of chest injury between the more violent Wenchuan earthquake and less violent Lushan earthquake, which affected similar regions of Sichuan province. Our analysis suggests that chest injuries occur more frequently in earthquake victims than previously thought, and that seismic intensity affects causes of chest injury. On the other hand, seismic intensity may not affect types of chest injury. These results may help emergency response centers predict the kinds of patients that they will need to treat and thereby lead to more rational allocation of limited medical resources.

The fact that our hospital was the major provider of medical services to patients in both the Wenchuan and Lushan earthquakes gives us a unique opportunity to examine how seismic intensity may affect the prevalence and characteristics of different types of injuries. Normally such comparisons are difficult because the earthquakes being compared differ in numerous respects. In this case, however, both Lushan and Wenchuan counties have similar geologic features and are located at the Longmenshan Fault Zone [10]. Other factors known to affect earthquake losses, such as demographic structure, population density, outbreak time, architectural features and local medical infrastructure, are also similar between the two affected areas [11]. Our hospital is approximately equidistant (100 km) from both epicenters. Thus, the major difference between the two earthquakes in our analysis is seismic intensity, allowing us to draw conclusions about how this parameter may affect casualties admitted to hospital.

Chest injuries were diagnosed in 9.9% of the patients admitted to our hospital in the Wenchuan earthquake [4] and 22.2% in the Lushan earthquake. These incidences are consistent with reports suggesting a 10–15% incidence of chest injuries among trauma patients [12], [13]. At the same time, most chest injuries in our patients were minor or moderate and resolved after conservative treatment, as reported in other major earthquakes [2], [3], [14]. The exception to this, both in our study and previous work, is severe chest injury, especially crushing injuries of the chest, which are associated with high mortality [15]–[18].

Contrary to our expectations, the incidence of chest injury was higher among patients in the less violent Lushan earthquake than in the more violent Wenchuan disaster, as was the incidence of crushing and other severe chest injuries. In fact, the incidence of chest injury in Lushan is higher than that reported in previous studies of chest injuries in several major earthquakes [1], [3], [19]–[21]. We speculate that all these findings reflect the fact that major transportation networks were more severely disrupted in the Wenchuan disaster, significantly delaying the arrival of patients at our hospital and leading to a larger number of pre-admission deaths due to severe chest trauma. In the Lushan earthquake, in contrast, most transportation networks remained open, facilitating prompt rescue and delivery to our hospital. Therefore we suspect that the data on severe chest injury in our Lushan cohort more accurately reflect what happens in a major earthquake. Previous studies of severe chest injury and other types of severe injury in earthquakes should be interpreted with caution given the risk of underestimating prevalence as a result of missing individuals and pre-admission deaths. Our findings further suggest that prompt rescue and medical attention after destructive earthquakes may save the lives of many patients with severe chest injuries.

Our comparison of the two earthquakes identified several other informative differences. While injuries due to being accidentally struck were the most common type of injury in both earthquakes, injuries due to falls were more frequent in the less intense Lushan earthquake, while injuries due to being buried were more frequent in the more intense Wenchuan disaster. These results can be attributed to seismic intensity since more intense earthquakes like the Wenchuan disaster involve more extensive structural damage, leading to building collapse and increasing the risk of burial. In less intense earthquakes such as Lushan, fewer buildings collapse, so a greater proportion of injuries are due to falls. Only 4 aftershocks at >5.0 on the Richter scale were recorded in the Lushan earthquake, and all were nondestructive. This may explain why only 5 patients in our Lushan cohort (6.9% of the total admitted) were injured during aftershocks; most of these injuries were due to falling or being accidentally struck. These individuals may have been engaging in unsafe behaviors during the disaster, perhaps as a result of psychological or physical trauma after the primary shocks [22].

Our findings lead us to recommend the deployment of emergency responders trained in dealing with such critical conditions as crush syndrome, severe infection and brain injuries in the wake of violent earthquakes, where the risk of burial-related injuries is high. In less intense earthquakes, rapid response of psychologists may be particularly important for preventing avoidable injuries due to shock and disorientation.

The most frequent types of chest injury in both earthquakes were rib fractures and pulmonary contusions, similar to the causes of chest injuries in other medical emergencies [23]. Comparing the two earthquakes showed that greater seismic intensity was associated with greater prevalence of rib fractures but lower prevalence of pulmonary contusion. Greater intensity was also associated with higher frequencies of severe injuries like hemothorax and pneumothorax. These findings should be interpreted with caution given the limited sample size, due to lack of reliable data on where most patients sustained their injuries within the earthquake-affected areas. In fact, combining the results of Tables 2 and 5 suggests that seismic intensity may not affect the overall distribution of chest injury types. However, this analysis is based on a crude stratification of intensities into only two categories. Future work should involve many more patients and more detailed stratification of intensity. Despite these limitations, the data suggest that greater proportions of patients in more violent earthquakes may require closed drainage of the pleural cavity. Our lack of statistical power prevents us from drawing any conclusions about a possible relationship between seismic intensity and rare injuries such as diaphragmatic hernia and thoracic duct injury.

In earthquakes, patients presenting only with chest injury are rare; most chest injuries occur simultaneously with other types of injury [1], [3]. Similarly we found that 84.7% of Wenchuan chest injury patients and all Lushan chest injury patients presented concurrently with other types of injury. Since injuries that occur together with chest injuries can affect patient outcomes, we compared the prevalence and types of such concurrent injuries between the two earthquakes. In both disasters, injuries of the face, neck and abdomen were uncommon, and external injuries were frequent but usually mild (Table 3). More than 50% of Wenchuan patients admitted to our hospital [7] and more than 70% of Lushan patients were treated in the orthopedic department, similar to what has been reported in other earthquakes [24]. Injuries of the spine and extremities were the most frequent injuries concurrent with chest injuries. We were surprised to find that the prevalence of concurrent injuries affecting the brain, skull, spine or spinal cord was higher in the less violent Lushan earthquake. Again, the explanation may reflect the fact that many patients with severe injuries of the brain and spinal cord die rapidly, so a greater proportion of such patients in the Wenchuan earthquake may have died before they could be rescued and transported to our hospital. Therefore we recommend the deployment of adequate numbers of orthopedic and neurological specialists to attend to patients with chest injuries in violent earthquakes.

Accurately predicting which earthquake casualties are likely to suffer respiratory failure would significantly aid in emergency triage and allocation of limited medical resources. The three injury scales ISS, NISS, and CII, all based on the AIS2005 scale, are widely used to predict survival in disasters, and we recently applied them to the prediction of respiratory failure among Wenchuan chest injury patients [4]. Our results in that study suggested that the NISS was superior to the other scales. When we examined the Lushan cohort, however, all three scales showed unsatisfactory AUC values ranging from 0.590–0.720, compared to values of 0.837–0.893 in the Wenchuan earthquake [4]. While the high sensitivity of ISS may make it useful as a screening method, NISS and CII show poor sensitivity and moderate specificity, limiting their diagnostic usefulness. The AIS2005 scale assesses only injury severity, regardless of how long the patient has had the injury and what treatments have been administered [25]. Nevertheless, clinicians often use it to predict trauma progression [1], [26]–[28], even though many factors other than injury severity affect such progression. In earthquake-related trauma in particular, factors such as pre-admission treatments, the delay between injury and treatment, and the presence of comorbidities may also affect trauma progression. All these factors may therefore influence the risk of respiratory failure, so we recommend combining conventional injury severity scales with more comprehensive trauma assessment in order to predict respiratory failure in chest injury patients.

Our analysis of patients admitted to our hospital in the Wenchuan and Lushan earthquakes leads us to several conclusions. Previous studies may have underestimated the true incidence of chest injuries in violent earthquakes, and these injuries most frequently involve rib fractures or pulmonary contusions due to being accidentally struck. Greater seismic intensity is associated with lower prevalence of injuries due to falling and higher prevalence of injuries due to being buried. However, seismic intensity does not appear to affect the prevalence and types of chest injury. Earthquake-related chest injuries usually occur together with other injuries, most frequently with orthopedic injuries. The prevalence and types of concurrent injuries may be affected by the delay between rescue and treatment as well as the severity of the earthquake. Injury severity scales by themselves are inadequate for predicting respiratory failure in chest injury patients in earthquakes; more factors should be taken into account when evaluating such trauma.

## Supporting Information

Data S1 **Raw data of chest injury characteristics and concurrent injuries in patients admitted to our hospital in the Wenchuan and Lushan earthquakes.**
(XLS)
